# Supplementary material for: Modeling glioblastoma heterogeneity as a dynamic network of cell states
Source: Mol Syst Biol. 2021 Sep 16;17(9):e10105. doi: 10.15252/msb.202010105 (PMC8444284; doi:10.15252/msb.202010105)
Supplement: Supplementary file 5 — Source Data for Figure 3 [file MSB-17-e10105-s001.zip › Figure3A_sourcedata/GSEA_3065/nosigdb_state1.GseaPreranked.1622718409535/POLLEN_OUTERRADIALGLIA_PMID_26406371.html]

Details for gene set POLLEN\_OUTERRADIALGLIA\_PMID\_26406371[GSEA]

|  || Dataset | state1 |
| Phenotype | NoPhenotypeAvailable |
| Upregulated in class | na\_neg |
| GeneSet | POLLEN\_OUTERRADIALGLIA\_PMID\_26406371 |
| Enrichment Score (ES) | -0.754993 |
| Normalized Enrichment Score (NES) | -2.3441846 |
| Nominal p-value | 0.0 |
| FDR q-value | 0.0 |
| FWER p-Value | 0.0 |
Table: GSEA Results Summary

  

Fig 1: Enrichment plot: POLLEN\_OUTERRADIALGLIA\_PMID\_26406371      
 Profile of the Running ES Score & Positions of GeneSet Members on the Rank Ordered List

  

| PROBE | GENE SYMBOL | GENE\_TITLE | RANK IN GENE LIST | RANK METRIC SCORE | RUNNING ES | CORE ENRICHMENT || 1 | LGALS3 |  |  | 117 | 0.348 | 0.0176 | No |
| 2 | GNG5 |  |  | 276 | 0.269 | 0.0244 | No |
| 3 | NOG |  |  | 381 | 0.231 | 0.0334 | No |
| 4 | RGS16 |  |  | 499 | 0.206 | 0.0389 | No |
| 5 | CDK6 |  |  | 999 | 0.134 | -0.0005 | No |
| 6 | MOXD1 |  |  | 1104 | 0.122 | -0.0007 | No |
| 7 | CARHSP1 |  |  | 2827 | 0.030 | -0.1733 | No |
| 8 | GULP1 |  |  | 3139 | 0.022 | -0.2031 | No |
| 9 | CDCA7L |  |  | 3390 | 0.016 | -0.2272 | No |
| 10 | EEPD1 |  |  | 3518 | 0.013 | -0.2390 | No |
| 11 | FHOD3 |  |  | 3786 | 0.008 | -0.2654 | No |
| 12 | ETV5 |  |  | 3894 | 0.006 | -0.2758 | No |
| 13 | MGST3 |  |  | 4220 | 0.000 | -0.3088 | No |
| 14 | STOM |  |  | 4388 | -0.003 | -0.3256 | No |
| 15 | BMP7 |  |  | 4562 | -0.006 | -0.3427 | No |
| 16 | SEL1L3 |  |  | 4651 | -0.007 | -0.3511 | No |
| 17 | TCF7L2 |  |  | 4707 | -0.008 | -0.3560 | No |
| 18 | FBN2 |  |  | 4789 | -0.009 | -0.3635 | No |
| 19 | RHOJ |  |  | 5068 | -0.014 | -0.3907 | No |
| 20 | IDI1 |  |  | 6154 | -0.032 | -0.4983 | No |
| 21 | FUT8 |  |  | 6330 | -0.035 | -0.5131 | No |
| 22 | PAQR8 |  |  | 7316 | -0.058 | -0.6084 | No |
| 23 | DOCK1 |  |  | 7505 | -0.064 | -0.6221 | No |
| 24 | HOPX |  |  | 7850 | -0.075 | -0.6507 | No |
| 25 | SOAT1 |  |  | 8031 | -0.082 | -0.6620 | No |
| 26 | RAB3GAP2 |  |  | 8198 | -0.089 | -0.6714 | No |
| 27 | ARAP2 |  |  | 8907 | -0.134 | -0.7320 | No |
| 28 | MT3 |  |  | 8954 | -0.139 | -0.7249 | No |
| 29 | LIFR |  |  | 9216 | -0.168 | -0.7372 | No |
| 30 | PMP22 |  |  | 9392 | -0.199 | -0.7381 | Yes |
| 31 | TFPI |  |  | 9427 | -0.206 | -0.7240 | Yes |
| 32 | FKBP9 |  |  | 9445 | -0.211 | -0.7078 | Yes |
| 33 | LGALS3BP |  |  | 9447 | -0.212 | -0.6900 | Yes |
| 34 | IL6ST |  |  | 9519 | -0.231 | -0.6777 | Yes |
| 35 | BRCA1 |  |  | 9600 | -0.258 | -0.6639 | Yes |
| 36 | TNC |  |  | 9605 | -0.262 | -0.6421 | Yes |
| 37 | TMEM132B |  |  | 9613 | -0.264 | -0.6205 | Yes |
| 38 | APBB2 |  |  | 9645 | -0.277 | -0.6001 | Yes |
| 39 | ABAT |  |  | 9646 | -0.278 | -0.5765 | Yes |
| 40 | MLC1 |  |  | 9728 | -0.338 | -0.5561 | Yes |
| 41 | ITM2C |  |  | 9758 | -0.363 | -0.5283 | Yes |
| 42 | ETV1 |  |  | 9764 | -0.371 | -0.4973 | Yes |
| 43 | SAT1 |  |  | 9807 | -0.445 | -0.4639 | Yes |
| 44 | EDNRB |  |  | 9824 | -0.509 | -0.4223 | Yes |
| 45 | FABP7 |  |  | 9825 | -0.513 | -0.3788 | Yes |
| 46 | FGFBP3 |  |  | 9850 | -0.651 | -0.3260 | Yes |
| 47 | SEMA5A |  |  | 9860 | -0.749 | -0.2635 | Yes |
| 48 | PTPRZ1 |  |  | 9869 | -0.918 | -0.1864 | Yes |
| 49 | TTYH1 |  |  | 9872 | -1.033 | -0.0989 | Yes |
| 50 | GFAP |  |  | 9877 | -1.174 | 0.0002 | Yes |
Table: GSEA details [plain text format]

  

Fig 2: POLLEN\_OUTERRADIALGLIA\_PMID\_26406371: Random ES distribution      
 Gene set null distribution of ES for **POLLEN\_OUTERRADIALGLIA\_PMID\_26406371**
